# Metallic glass nanostructures of tunable shape and composition

**DOI:** 10.1038/ncomms8043

**Published:** 2015-04-22

**Authors:** Yanhui Liu, Jingbei Liu, Sungwoo Sohn, Yanglin Li, Judy J. Cha, Jan Schroers

**Affiliations:** 1 Center for Research on Interface and Surface Phenomena, Yale University, New Haven, Connecticut 06520, USA; 2 Department of Mechanical Engineering and Materials Science, Yale University, New Haven, Connecticut 06520, USA; 3 Energy Sciences Institute, Yale West Campus, West Haven, Connecticut 06516, USA

## Abstract

Metals of hybrid nano-/microstructures are of broad technological and fundamental interests. Manipulation of shape and composition on the nanoscale, however, is challenging, especially for multicomponent alloys such as metallic glasses. Although top–down approaches have demonstrated nanomoulding, they are limited to very few alloy systems. Here we report a facile method to synthesize metallic glass nanoarchitectures that can be applied to a broad range of glass-forming alloys. This strategy, using multitarget carousel oblique angle deposition, offers the opportunity to achieve control over size, shape and composition of complex alloys at the nanoscale. As a consequence, nanostructures of programmable three-dimensional shapes and tunable compositions are realized on wafer scale for metallic glasses including the marginal glass formers. Realizing nanostructures in a wide compositional range allows chemistry optimization for technological usage of metallic glass nanostructures, and also enables the fundamental study on size, composition and fabrication dependences of metallic glass properties.

## Abstract (web summary)

The nanoscale manipulation of the shape and composition of metallic glasses is challenging. Here, the authors employ a multitarget carousel oblique angle deposition strategy to synthesize metallic glass nanoarchitectures, and demonstrate applicability to a range of glass-forming alloys.

## Graphical abstract

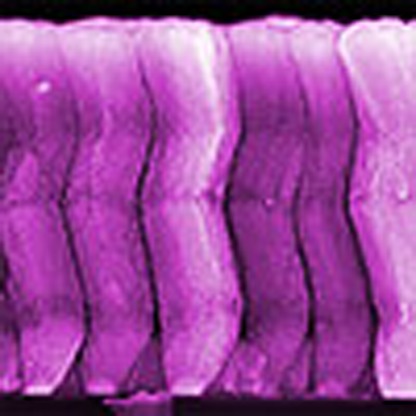

Nanoarchitectured metals with large surface-to-volume ratio are of broad technological and fundamental significance, particularly, for sensing, detecting and catalysis^1^. In the past decades, tremendous efforts have been made to improve their performance, and it has been realized that attributes of a material on the nanoscale depend on atomic structures, size, shape and chemistry^2^,^3^. The prerequisite for accurate fabrication and design of functionality on the nanoscale is that the building blocks must be smaller than the nanoscale structures^3^. This is a challenge for most material classes, but the case for metallic glasses. Exhibiting superior mechanical properties compared with conventional crystalline metals, metallic glasses are amorphous alloys that are homogeneous and isotropic from macro- down to nanoscale^4^,^5^,^6^,^7^. The wide recognition of metallic glasses as an ideal material for nanoscale applications explained the excitement about their first synthesis as nanorods^8^, which has triggered intensive research on advancing nanomoulding techniques^7^,^8^,^9^,^10^,^11^,^12^,^13^. Utilizing the thermoplastic-forming property of metallic glasses in their supercooled liquid regions and balancing with capillary forces^8^, nanoscaled metallic glasses (NMGs) have been obtained^8^,^9^,^10^ and often found to display drastically enhanced properties^7^,^11^,^12^,^13^.

For the successful synthesis of NMGs via nanomoulding, the metallic glass must possess a combination of specific properties such as sufficient wetting of the mould, quantified by a wetting angle of below 140°, low oxidation rate and a large thermoplastic-forming ability^8^. Such properties are required to fill nanoscale moulds of high aspect ratio before termination of this process by crystallization^14^. For the exploration of NMGs structures in sensing, catalytic or electrochemical applications, in addition to the above requirements, a specific and optimized chemistry is a prerequisite^11^. Hence, there is a demand for a fabrication technique that yields metallic glass nanostructures architecture in a broad and tunable range of compositions and geometry. Currently, however, only very few metallic glasses fulfil these requirements for the fabrication of NMGs.

Here we introduce a bottom-up method based on multitarget carousel oblique angle deposition (multi-COAD) to fabricate NMG architectures of tunable chemistry and controllable three-dimensional (3D) shapes. The versatility of this approach allows us to realize, at high yields, NMGs in a broad range of compositions.

## Results

### Fabrication principle

**Figure 1:**
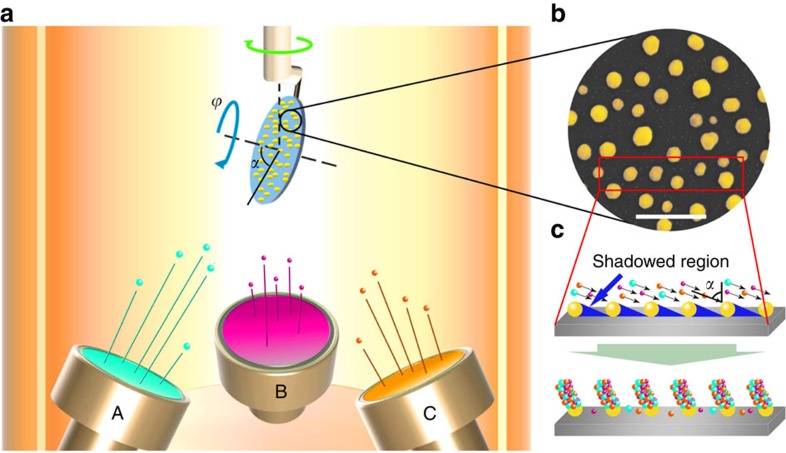
The scheme of multiple targets COAD. (**a**) Configuration of the deposition system. The sputtering guns are arranged in a confocal geometry, but the tilt angle of each gun can be changed independently. The angle-*α* between the normal of the substrate and the incoming flux is called glancing angle. Variation of azimuthal angle *ϕ* controls the structure uniformity and also allows the creation of complex structures. During deposition, the substrate spins along the direction of the green arrow. (**b**) Morphology of a template with ∼100 nm gold nanoparticles prepared by dewetting an ultrathin gold film. (**c**) Illustration of shadowed growth. Scale bar, 500 nm (**b**).

The multi-COAD method is illustrated in Fig. 1a. It combines multiple targets co-sputtering^15^,^16^,^17^ with oblique angle deposition^2^,^18^,^19^. In conventional deposition of metallic glasses, a single alloy target is typically used for sputtering. However, in many cases, the deposited composition differs significantly from that of the alloy target^20^, rendering the control of composition difficult. We demonstrated recently that co-sputtering using multiple elemental targets allows fine tuning of composition^17^. The high effective cooling rate on the order of 10^9^ K s^−1^ (ref. 21) during the deposition process enables the formation of glassy films in a wide composition range including the vast group of marginal metallic glass formers. This offers the opportunity to optimize chemistry tailored for desired NMG functions. In contrast to the usual oblique angle deposition^18^,^19^, which is also known as glancing angle deposition and has been utilized to produce nanoarchitectures of simple chemistry^22^,^23^, multi-COAD comprises three sputtering targets arranged in a confocal geometry. The substrate is orientated with a glancing angle, *α*, with respect to the targets. This angle can be manipulated independently relative to each sputtering target by changing either the substrate orientation or the tilt angle of the sputtering guns. Variation of the azimuthal angle, *ϕ*, primarily not only warrants the control of NMG uniformity but can also be utilized to achieve complex 3D shapes. Deposition with multi-COAD proceeds in a carousel fashion^24^, where the substrate holder rotates at constant speed (see the green arrow in Fig. 1a). After receiving the flux from target A during holder rotation, the substrate conforms to target B and subsequently to target C. The compositional homogeneity and glassy structure is controlled by varying independently the deposition rate from each target and the rotation speed of the carousel substrate holder^24^.

The first step in the bottom-up synthesis of NMGs via multi-COAD is the preparation of a template to catalyse the directional growth of the nanostructures. Various methods are established for template synthesis with either ordered or disordered nano- or micropatterns. Among those are optical or electron-beam lithography, nanocolloid self-assembly, embossing and block co-polymer micelle nanolithography^25^. Since our focus is to demonstrate the feasibility and universality of multi-COAD for the versatile synthesis of metallic glass nanoarchitectures, we fabricate for convenience the template by dewetting gold thin films on silicon wafer to yield Au nanoparticles^26^. Figure 1b shows such a template with Au nanoparticles of a mean size of ∼100 nm. The mean particle size can be controlled through the initial gold film thickness (Supplementary Fig. 1). When the template is exposed to vapour flux at a glancing angle, the nanoparticles act as seeds onto which the incoming atoms nucleate and grow in a columnar manner (Fig. 1c) owing to the amplified shadowing effect^2^.

### Wafer-scale synthesis of metallic glass nanostructures

**Figure 2:**
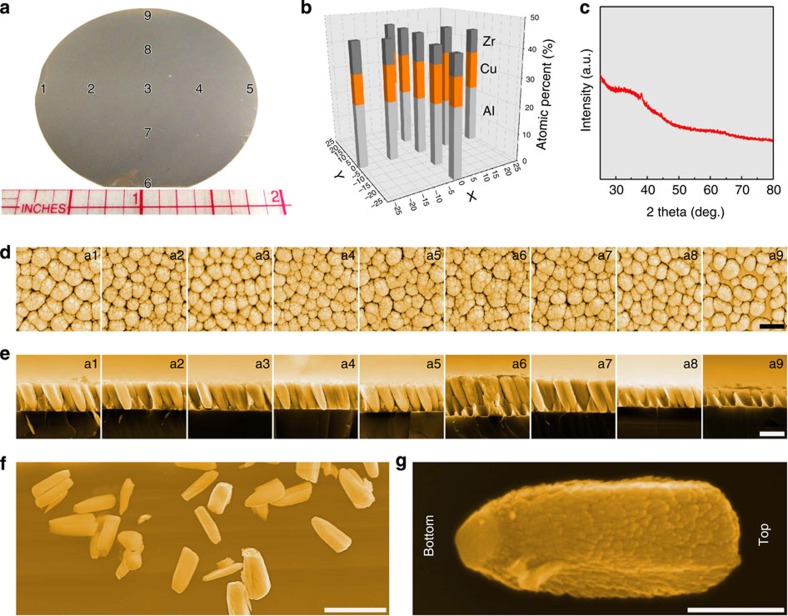
Grown metallic glass nanostructures. (**a**) Appearance of as-grown metallic glass nanostructures on a 50-mm-diameter Si wafer. (**b**) Energy dispersive X-ray analyses at different locations as labelled by the numbers indicate a homogeneous composition across the wafer. (**c**) XRD spectrum on the as-grown nanostructures. The halo pattern suggests the amorphous nature of the samples. The sharp Bragg peak originates from the gold template. (**d**) Top view and (**e**) side view of the morphologies of the metallic glass nanostructures at locations labelled by the numbers in **a**. (**f**) Free-standing nanorods. (**g**) Close-up of individual rods. Scale bars, 1 μm (**d**,**e**), 2 μm (**f**) and 200 nm (**g**).

To demonstrate multi-COAD, we choose ZrCuAl alloy system. Owing to its low formability and high oxidation rate, ZrCuAl NMGs have not been realized using nanomoulding technique. To demonstrate the versatility of our approach, using elemental sputtering targets, we synthesize NMGs with a composition that is far away from the best glass-forming alloy in this system. Figure 2a shows the ZrCuAl NMGs on a 50-mm-diameter Si wafer templated with ∼50 nm gold nanoparticles. Energy dispersive X-ray analysis at different locations of the wafer (Fig. 2a) confirms the homogeneous chemistry across the entire wafer (Fig. 2b and Supplementary Table 1). The composition of the NMGs is Zr_44_Cu_35_Al_21_, which is nearly identical to the set composition of Zr_45_Cu_35_Al_20_ through deposition rate. This indicates that the composition of the NMG structures fabricated by multi-COAD can be controlled accurately. Figure 2c displays the X-ray diffraction (XRD) spectrum of the as-grown NMGs. The halo diffraction pattern centred ∼36° confirms the amorphous structure of the NMGs. The weak Bragg peaks in the spectrum originate from the gold nanoparticles used as template.

Figure 2d,e displays the NMG morphologies observed at different locations of the wafer, top and side views, respectively. The NMGs are rods with uniform lateral dimensions. Originating from the oblique deposition angle, the rods are tilted by ∼20° relative to the substrate normal (Fig. 2e), which is dependent on the azimuthal angle-*ϕ*, the glancing angle-*α* and deposition rate, all of which are tunable^19^. Figure 2f presents the free-standing nanorods. The NMGs, as shown in Fig. 2g, exhibit surfaces that are decorated with small features. The surface of multi-COAD-synthesized NMGs can be controlled by the deposition conditions, such as sputtering gas pressure^20^. This enhanced surface area is beneficial for applications, such as surface-enhanced Raman scattering catalysis^1^. Usually, NMGs produced by thermoplastic moulding have smooth surfaces^8^,^10^. For an increased surface area, additional processing steps are needed, which, however, sacrifice the glassy structures^27^,^28^. The multi-COAD-grown hierarchical nanostructures suggest that further processing steps to enhance surface area are not required, so that the advantages of the amorphous structure can be fully retained.

Along the horizontal direction in Fig. 2a, such as from 1 to 5, the rods exhibit similar lengths. A gradual decrease in rod length can be seen along the vertical direction, for instance, from location 6 to 9. This length variation relative to the distance from the sputtering target is a consequence of the deposition rate difference at a constant azimuthal angle*-ϕ*^19^. Variation of *ϕ* can lead to improved length uniformity^19^. The high number density of NMGs is set by the template choice, here, the dewetted gold nanoparticles for seeding (Fig. 2e). Generally, the spacing and arraying of the rods can be controlled by tailoring the seeding templates as demonstrated with other materials^29^.

### Metallic glass nanoarchitecture of complex 3D shape

**Figure 3:**
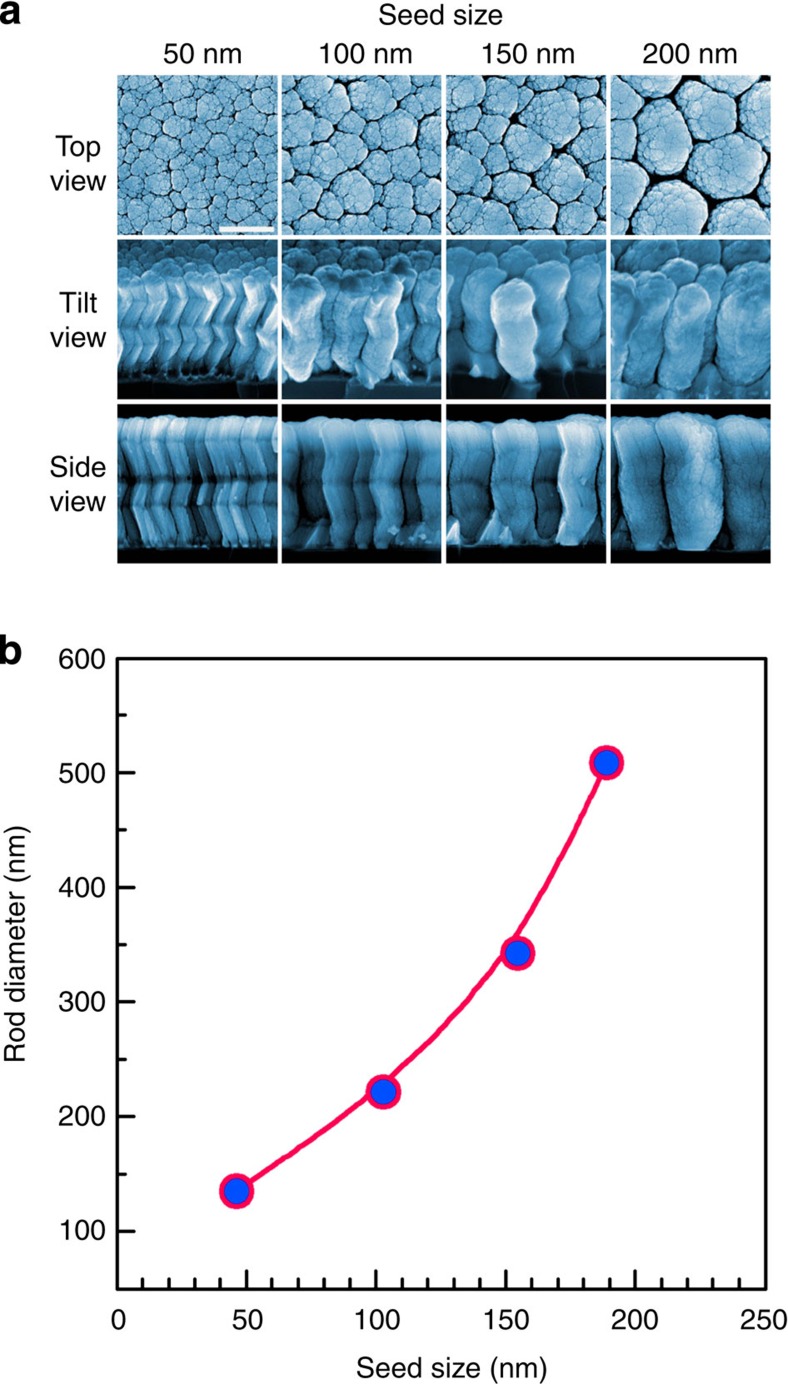
Seed size controlled metallic glass nanostructures of complex shapes. (**a**) Zigzag nanostructures made from ZrCuAl metallic glass on templates of different nanoparticles. (**b**) Plot of the lateral dimensions of the nanostructures as function of template size. Scale bar, 500 nm (**a**).

The unique thermoplastic formability of metallic glasses has been explored to shape complex geometries on the macroscale, of which many were predominately unachievable with other metals^30^. In contrast on the nanoscale, only simple shapes, such as dots, rods or trenches, can be fabricated using moulding^8^,^9^,^10^,^31^. Complex nanoscale shapes of large aspect ratio are unachievable due to wettability requirements of a metallic glass with mould and the unavailability of complex moulds. During multi-COAD, the variation of *ϕ*, which is usually used to improve uniformity, enables us to manipulate the shape of the NMGs in three dimensions^19^. Figure 3a shows zigzag structures made from ZrCuAl growing on templates of various seed sizes. The zigzag structures are fabricated by periodically changing the template orientation (for example, *ϕ*=180°) around its normal. The zigzag-shaped NMGs can be grown to a large aspect ratio. In Fig. 3a, an aspect ratio of ∼20 is obtained for the NMGs grown on the smallest seeds. Even larger aspect ratios can be achieved by a prolonged deposition time for each segment or increased number of segments. The angles between the segments of the zigzag structures can be altered through the variation of glancing angle, *α*^32^,^33^. As shown in Fig. 3b, the lateral dimension of the structures is dependent on the size of seeding particles in the template, suggesting that it is possible to modulate the dimension of the NMGs by varying the seed size. Although the smallest seed size is ∼50 nm in this work, smaller dimensions can be achieved using smaller seeds^2^,^29^. These grown zigzag NMGs of high aspect ratios are only one example of the geometrical versatility of multi-COAD.

### Hybrid metallic glass nanostructures

**Figure 4:**
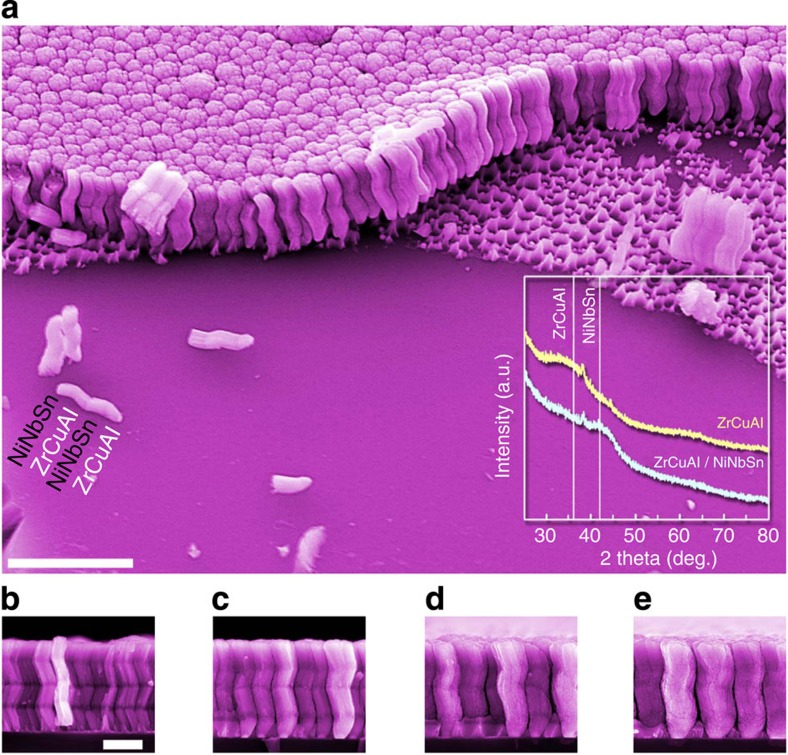
Hybrid nanostructures comprising two metallic glasses. (**a**) The overview of large area showing the morphologies of the hybrid nanostructures, the shadowed growth and dispersed individual zigzag rods. The inset shows the XRD spectra of nanostructures consisting of single ZrCuAl glass phase and hybrid ZrCuAl/NiNbSn glass phases. The peaks in the spectra are due to gold from the templates. (**b**–**e**) Hybrid nanostructures of different lateral dimensions. Scale bars, 2 μm (**a**) and 500 nm (**b**-**e**).

To further demonstrate the versatility of multi-COAD, we fabricate hybrid nanoarchitectures (Fig. 4). The bottom-up approach offers the opportunity to create complex hybrid nanoarchitectures if the composition of the vapour flux is changed while growing. Figure 4a presents a hybrid structure comprising two metallic glasses, ZrCuAl and NiNbSn, grown on 100 nm seeding particles. Because of their low thermoplastic processability, both alloy systems are difficult to work with using the nanomoulding technique. With multi-COAD, however, we can vary the vapour chemistry from ZrCuAl to NiNbSn every time the template is reorientated around its normal. As a consequence, each segment contains different alloy materials (Fig. 4a). The inset of Fig. 4a displays the XRD spectra of ZrCuAl and ZrCuAl/NiNbSn NMG structures, respectively. The dual glassy diffuse peaks as indicated by the vertical lines in the spectrum of ZrCuAl/NiNbSn NMG confirm that the hybrid structures are composed of amorphous ZrCuAl and amorphous NiNbSn. As shown in Fig. 4b–e, despite the change in vapour chemistry during growth, we are still able to maintain the shape and lateral dimension of the structures as shown in Fig. 3a. To our knowledge, this is the first demonstration of metallic glass hybrid nanostructures, which allow integration of multiple components with different functionalities as demanded in sensing and detecting devices^34^.

### Nanoporous metallic glass membrane

**Figure 5:**
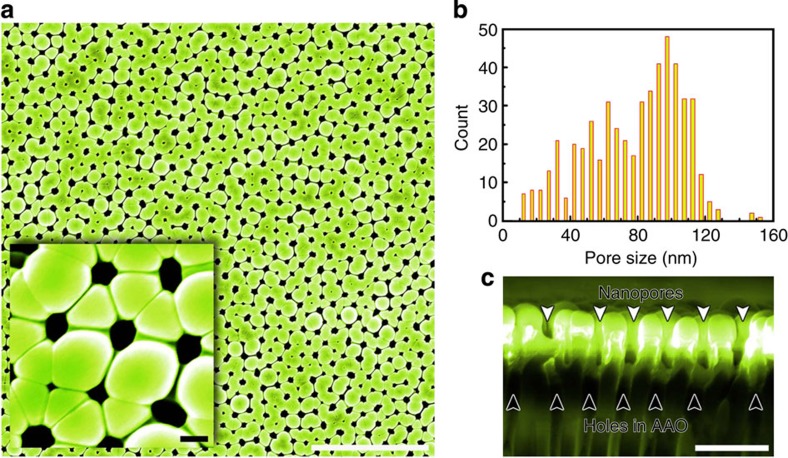
Nanoporous metallic glass membrane. (**a**) Top view of the porous MgCuY metallic glass membrane with through nanopores. The inset shows the close-up of the nanopores. (**b**) Distribution of pore size analysed with the image shown in **a**. (**c**) Side view of the membrane grown on an anodic aluminium oxide template with channel size of 100 nm. The size of the pore in the membrane is nearly the same as that of the channel in template. Scale bars, 2 μm (**a**), 100 nm (**a**, inset) and 500 nm (**c**). AAO, anodic alumina oxide.

In addition to the 1D nanostructures, multi-COAD can also synthesize 2D structures. Metallic glass nanoporous membranes are desired owing to their extraordinary strength and elasticity^5^,^7^. Fabrication of metallic glass nanoporous membranes, however, is difficult with existing methods. In contrast, our bottom-up synthesis through multi-COAD enables us to make metallic glass membranes (Fig. 5a) without the requirement for material removal as in thermoplastic moulding^7^,^30^. The nanoporous membrane shown in Fig. 5 is Mg_65_Cu_25_Y_10_ and was formed using the anodic alumina oxide as a template. The pores exhibit a mean size of ∼100 nm (Fig. 5b), the same size as the holes in the template (Fig. 5c). This indicates accurate replication of the template patterns using multi-COAD, and the porosity can be controlled by varying the feature sizes in the template. This process is distinct from typical deposition where flux impings parallel to substrate normal, which can fill the pores and result in a continuous film. We emphasize that hybrid membranes composed of different materials can be readily fabricated through multi-COAD by changing vapour chemistry during growth, the same strategy as in Fig. 4, and would, for example, yield different surface chemistries on the two sides of the membrane.

## Discussion

The multi-COAD approach demonstrates the facile fabrication of a wide variety of metallic glass nanoarchitectures of complex 3D shapes and hybrid chemistry. Unlike previous techniques to fabricate NMGs, our strategy is independent of the specific metallic glass properties such as glass-forming ability, thermal stability in the supercooled liquid region, wettability, and oxidation behaviour. Our approach can be readily extended to virtually all alloys that form an amorphous structure during deposition. Furthermore, using alloy targets in multi-COAD will enable synthesis of metallic glass nanoarchitectures composing >3 constituent elements and incorporation of elements of high vapour pressure such as phosphorus. We believe that the versatility to simultaneously control size, shape and chemistry using multi-COAD in combination with our high-throughput materials discovery^17^ will greatly broaden the exploration, in an unprecedented compositional space, of novel NMGs of desired function. From a scientific point of view, multi-COAD will enable studying the effect of chemistry, dimension and fabrication rate on glass formation and metallic glass properties.

## Methods

### Template preparation

The nanoparticle-decorated templates were fabricated by dewetting thin gold films. Silicon wafers of diameter of 50 mm were used as substrates for deposition. The films were prepared by DC magnetron sputtering of 50-mm-diameter gold target. The thickness of the gold film ranging from 5 to 20 nm was controlled by time on the base of deposition rate measured with a quartz crystal thickness monitor. Nanoparticles were obtained by annealing the as-deposited films at 500 °C for 30 min. The morphologies and size distributions of the nanoparticles are shown in Supplementary Fig. 1

### Material fabrication and characterization

The metallic glass nanostructures were grown using multi-COAD illustrated in Fig. 1. Before sputtering, the chamber was pumped down to a vacuum level better than 10^−6^ Pa. The working pressure was kept at 0.3 Pa during deposition. In this work, all samples were fabricated by DC magnetron sputtering at room temperature. The compositions of the deposited samples were controlled by independently controlling sputtering powers applied on each target. The carousel substrate holder was rotated at a speed of 80 r.p.m., which guaranteed the chemical homogeneity of NMGs. The length or thickness of the NMG structures were controlled by deposition time based on the deposition rate measurements with a quartz crystal thickness monitor. The amorphous nature of the as-fabricated nanostructures was characterized using a Rigaku SmartLab X-ray diffractometer with a Cu K*α* radiation source. The morphologies of the NMGs were observed using a Hitachi SU-70 scanning electron microscope equipped with field-emission gun. Cross-section observations were conducted by cleaving the wafers. Chemistry of the as-grown NMGs on Si wafer was measured using energy dispersive X-ray analysis attached to the scanning electron microscope.

## Author contributions

Y.H.L. and J.S. conceived the research. Y.H.L. conducted the experiments with help from J.L., S.S., Y.L.L. and J.J.C.. Y.H.L. and J.S. wrote the manuscript. All authors discussed and commented on the manuscript.

## Additional information

**How to cite this article:** Liu, Y. H. *et al*. Metallic glass nanostructures of tunable shape and composition. *Nat. Commun*. 6:7043 doi: 10.1038/ncomms8043 (2015).

## Supplementary Material

Supplementary Information Supplementary Figure 1 and Supplementary Table 1
