# Supplementary material for: Metallic glass nanostructures of tunable shape and composition
Source: Nat Commun. 2015 Apr 22;6:7043. doi: 10.1038/ncomms8043 (PMC4421810; doi:10.1038/ncomms8043)
Supplement: Supplementary Information — Supplementary Figure 1 and Supplementary Table 1 [file ncomms8043-s1.pdf]

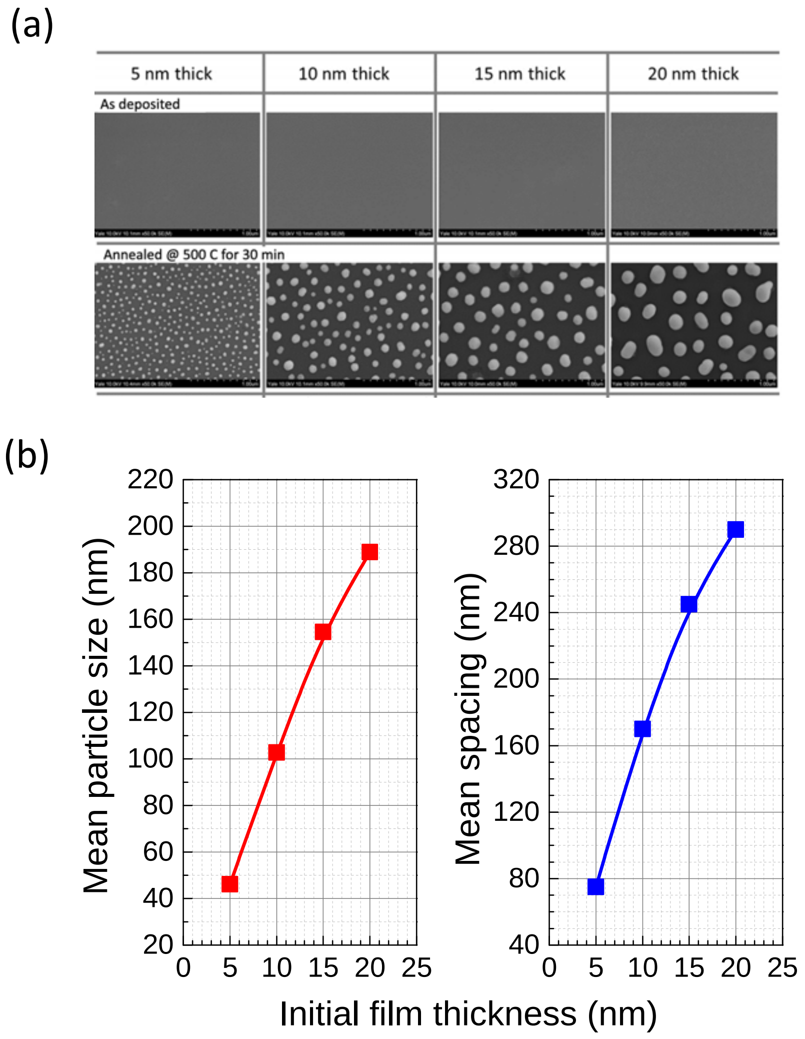

Supplementary Figure 1 (a) The morphologies of the as deposited gold thin films (upper row), the nanoparticles after annealing the films (middle and lower rows). (b) Dependence of mean particle size, mean spacing on initial film thickness.

Supplementary Table 1 Compositions of the NMGs shown in Fig. 2b, measured by using SEM/EDS.

Location	Zr (at.%)	Cu (at.%)	Al (at.%)
1	44	33.2	22.8
2	43.5	34.8	21.7
3	44.5	34.5	21.0
4	45.5	36.3	18.1
5	43.6	35.1	21.3
6	41.2	34.2	24.6
7	42.0	35.7	22.3
8	44.8	34.8	20.4
9	45.0	33.8	20.4
